# Crystal structure and Hirshfeld surface analysis of the naturally occurring cassane-type diterpenoid, 6β-cinnamoyl-7β-hy­droxy­vouacapen-5α-ol

**DOI:** 10.1107/S2056989018002499

**Published:** 2018-02-23

**Authors:** K. Osahon Ogbeide, Rajesh Kumar, Mujeeb-Ur-Rehman, Bodunde Owolabi, Abiodun Falodun, M. Iqbal Choudhary, Sammer Yousuf

**Affiliations:** a Department of Chemistry, Faculty of Physical Sciences, University of Benin, Benin City, Nigeria; b H. E. J. Research Institute of Chemistry, International Center for Chemical and Biological Sciences, University of Karachi, Karachi-75270, Pakistan; c Department of Chemistry, School of Sciences, The Federal University of Technology, Akure, Nigeria; d Department of Pharmaceutical Chemistry, Faculty of Pharmacy, University of Benin, Benin City, Nigeria

**Keywords:** crystal structure, *Caesalpinia pulcherrima*, cassane-type diterpenoids, Hirshfeld surface analysis, electrostatic potential

## Abstract

The title compound, C_29_H_36_O_5_, a cassane-type diterpenoid {systematic name: (4a*R*,5*R*,6*R*,6a*S*,7*R*,11a*S*,11b*R*)-4a,6-dihy­droxy-4,4,7,11b-tetra­methyl-1,2,3,4,4a,5,6,6a,7,11,11a,11b-dodeca­hydro­phenanthro[3,2-*b*]furan-5-yl 3-phenyl­prop-2-enoate}, was isolated from a medicinally important plant, *Caesalpinia pulcherrima* (Fabaceae). In the mol­ecule, three cyclo­hexane rings are *trans*-fused and adopt chair, chair and half-chair conformations. In the crystal, mol­ecules are linked *via* O—H⋯O hydrogen bonds, forming a tape structure along the *b-*axis direction. The tapes are further linked into a double-tape structure through C—H⋯π inter­actions. The Hirshfeld surface analysis indicates that the contributions to the crystal packing are H⋯H (65.5%), C⋯H (18.7%), O⋯H (14.5%) and C⋯O (0.3%).

## Abstract 2

The title compound, a cassane-type diterpenoid, was isolated from a medicinally important plant, *Caesalpinia pulcherrima* (Fabaceae). In the mol­ecule, the three cyclo­hexane rings are *trans*-fused and adopt chair, chair and half chair conformations.

## Chemical context   

*Caesalpinia pulcherrima* (Fabaceae) is a decorative evergreen plant widely used for the treatment of various illnesses (Roach *et al.*, 2003 ▸). It is commonly known as Gulmohor, Krishnachura and Mayirkonnai, respectively, in Hindi, Bengali and Tamil. Herbalists in the Amazon tropical rain forest have long known some of the medicinal uses of *C. pulcherrima*, known locally as ayoowiri (Patel *et al.*, 2010 ▸). The plant is also known to be used for the treatment of inflammation, earache, muscular and sore pain and cardiovascular disorders and as an anti­malarial, vermifuge and anti­pyretic agent (Patel *et al.*, 2010 ▸; Roach *et al.*, 2003 ▸). The natural constituents commonly known as cassane-type diterpenoids extracted from *C. pulcherrima* have been reported by Pranithanchai *et al.* (2009 ▸) and Rao *et al.* (2005 ▸). Cassane-type diterpenoids represent a class of pharmaceutically important natural products having various biological activities. The current study deals with the isolation, single-crystal X-ray diffraction and Hirshfeld surface analysis of the title compound, a naturally occurring cassane-type diterpenoid.

## Structural commentary   

**Figure 1:**
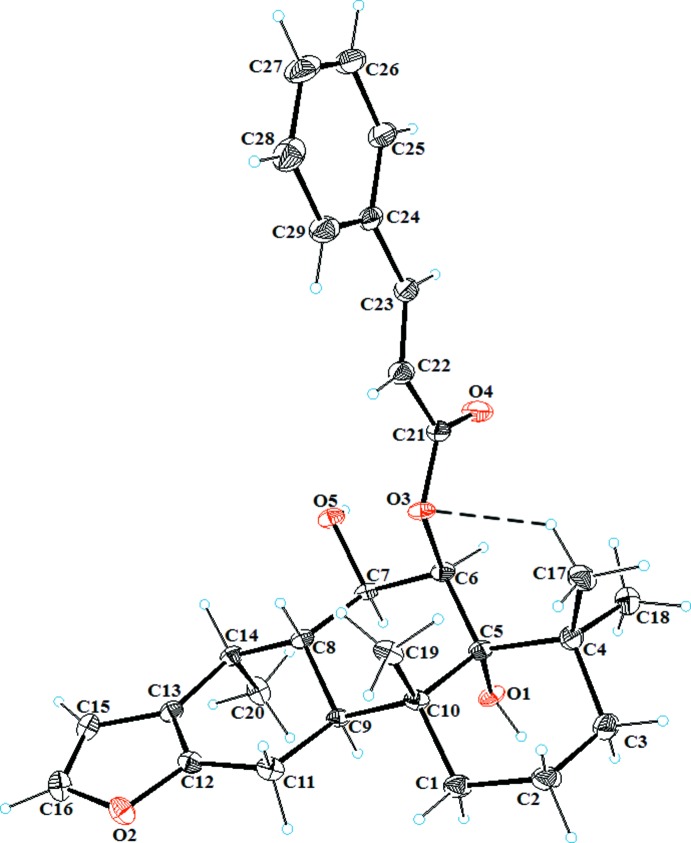
The mol­ecular structures of the title compound, showing atom-labelling scheme with displacement ellipsoids drawn at the 50% probability level. The intra­molecular C—H⋯O inter­action is shown as a dashed line.

The title compound is composed of three *trans*-fused cyclo­hexane rings, *A* (C1–C5/C10), *B* (C5–C10) and *C* (C8/C9/C11–C14), having chair, chair and half-chair conformations, respectively; the puckering parameters are *Q* = 0.561 (3) Å, θ = 0.0 (3)° and φ = 300 (132)° for ring *A*, *Q* = 0.555 (2) Å, θ = 4.4 (2)° and φ = 319 (4)° for ring *B*, and *Q* = 0.456 (2) Å, θ = 45.9 (3)° and φ = 17.7 (4)° for ring *C* (Fig. 1 ▸). The fused rings have *trans-*oriented hydroxyl and methyl groups attached at atoms C5 and C10, respectively, along the junction of rings *A* and *B*, with an O1—C5—C10—C19 torsion angle of −174.41 (18)°. The furan (O2/C12/C13/C15/C16) ring is essentially planar with the C12=C13 and C15=C16 double bonds having the same length (1.343 Å). The dihedral angle between the furan ring and the phenyl C24–C29 ring of the cinnamoyl moiety is 82.14 (13)°. The absolute configurations of the stereogenic centers at positions 5, 6, 7, 8, 9, 10 and 14 are established as *R*, *R*, *R*, *S*, *S*, *R* and *R*, respectively, on the basis of the reported structure by Fun *et al.* (2010 ▸). In the mol­ecule, an intra­molecular C—H⋯O inter­action (C17—H17*C*⋯O3; Table 1 ▸) forms an *S*(6) ring motif.
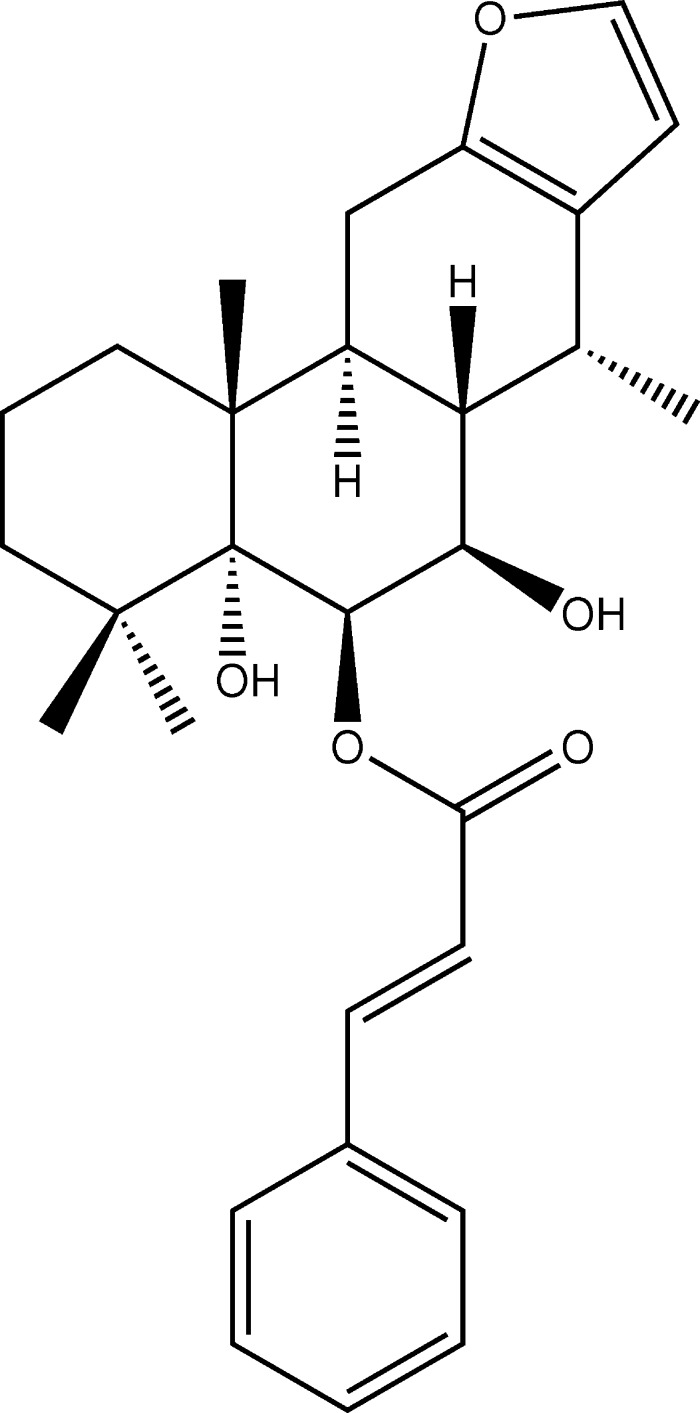


## Supra­molecular features   

**Figure 2:**
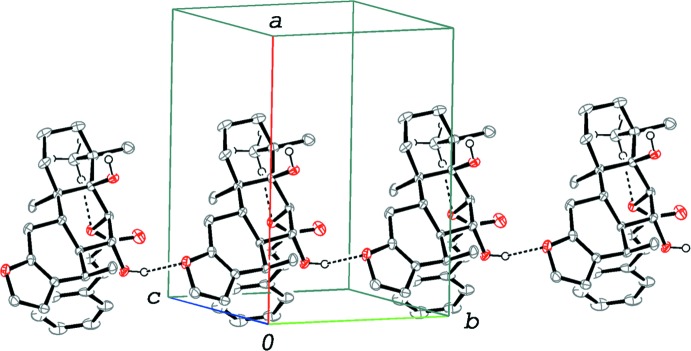
A packing diagram of the title compound. The O—H⋯O and C—H⋯O inter­actions are shown as dashed lines. H atoms except for the methyl group involved in the C—H⋯O hydrogen bond and the OH groups have been omitted.

In the crystal, the mol­ecules are linked *via* O—H⋯O hydrogen bonds (O5—H5⋯O2^i^; symmetry code as in Table 1 ▸), forming chains along the *b-*axis direction (Fig. 2 ▸). The chains are further linked into a double-chain structure through C—H⋯π inter­actions (C3—H3*A*⋯*Cg*1^ii^; symmetry code as in Table 1 ▸) involving the furan ring.

## Hydrogen bonding and Hirshfeld surface analysis   

**Figure 3:**
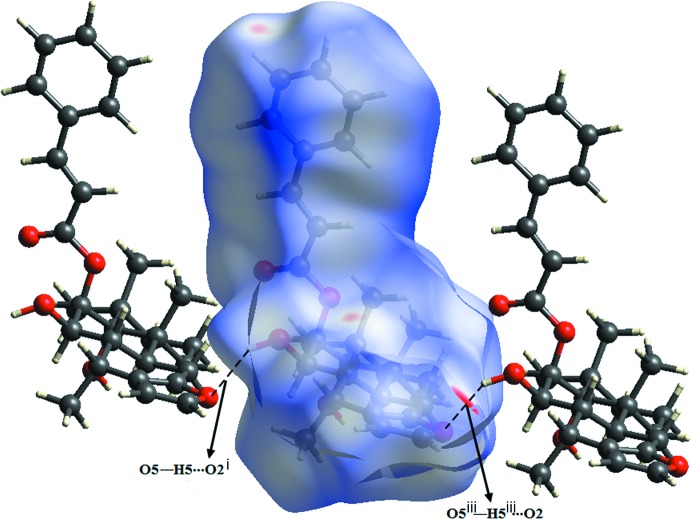
Hirshfeld surface over *d*_norm_ generated for the title compound and neighbouring mol­ecules linked *via* O—H⋯O hydrogen bonds (dashed lines). [Symmetry codes: (i) *x*, *y* + 1, *z*; (iii) *x*, *y* − 1, *z*.]

**Figure 4:**
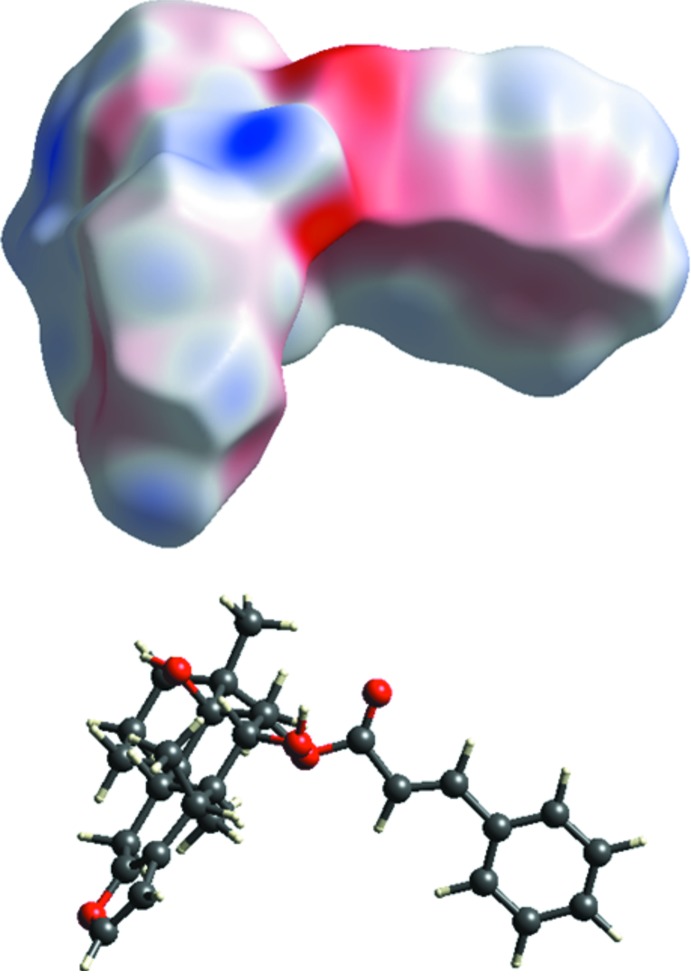
Electrostatic potential surface generated incorporated with Hirshfeld surface for compound (I).

**Figure 5:**
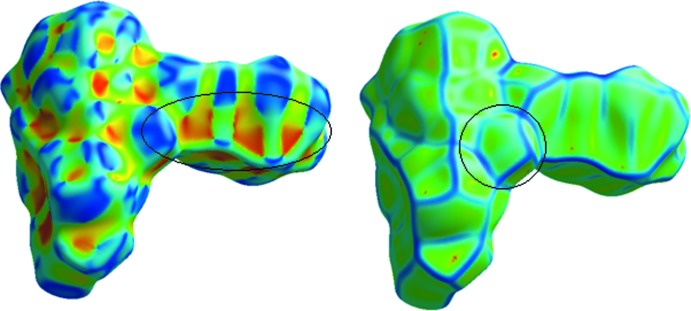
Hirshfeld surface mapped over shape-index calculated for the title compound.

**Figure 6:**
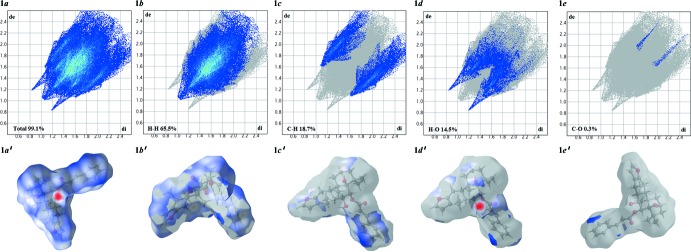
Two-dimensional fingerprint plots for compound (I).

The Hishfeld surface mapped over *d*_norm_ (McKinnon *et al.*, 2004 ▸; Spackman & Jayatilaka, 2009 ▸) for the title compound is depicted in Fig. 3 ▸. The red areas on the surface indicate short contacts as compared to the sum of the Van der Waals (vdW) radii, while the blue indicate long contacts and white area indicate contacts with distances equal to the sum of the vdW radii. The red highlighted area shows the O—H⋯O hydrogen bond, which is responsible for connecting mol­ecules to each other. The contribution of the H⋯H contacts to the crystal packing is 65.5%, and C⋯H, O⋯H and C⋯O contributions are 18.7, 14.5 and 0.3%, respectively. The Hirshfeld surface mapped over electrostatic potential (Spackman *et al.*, 2008 ▸) is shown in Fig. 4 ▸. The red region indicates atoms having potential to be hydrogen-bond acceptors with negative electrostatic potential, while the blue shows potential to be hydrogen-bond donors with positive electrostatic potential. Fig. 5 ▸ shows the Hishfeld surface mapped over shape-index and two-dimensional fingerprint plots are given in Fig. 6 ▸.

## Database survey   

A search of the Cambridge Structural Database (Version 5.38; Groom *et al.*, 2016) for a common fragment composed of three *trans-*fused six-membered rings and one planar furan ring shows 12 hits: Refcodes CSLPIN10 (Birnbaum *et al.*, 1969 ▸), DUTJIM, DUVCON (Fun *et al.*, 2010 ▸), EGAYIU, EGAYUG, EGAZAN, and EGAZER (Jiang *et al.*, 2002 ▸), MEYREN, MEYRIR, MEYROX and MEYRUD (Jiang *et al.*, 2001 ▸), and POPNIR (Kitagawa *et al.*, 1994 ▸). All of the hits are of the same class of compounds as the title compound, *i.e*. cassane-type diterpenoids, with different substitution patterns on the fused rings.

## Isolation and crystallization   

The powdered stem bark (2.5 kg) of *C. pulcherrima* was extracted with methanol (7.5 l) by cold maceration for four days, followed by filteration and concentration using a rotary evaporator under reduced pressure at 228 K to obtain the crude plant extract (200 g). The crude extract was further fractionated by silica gel chromatography first using *n*-hexane (9.4 l) and then with increasing polarities of solvents [*n*-hexa­ne:ethyl­acetate (1:1) (12.5 l), ethyl acetate (8.2 l), ethyl acetate:methanol (1:1) (13 l) and finally with methanol (7 l)]. Concentration of fractions *in vacuo* gave five major fractions of 0.45, 38.81, 25.75, 127.73 and 4.18 g after elution from *n*-hexane, *n*-hexa­ne:ethyl acetate (1:1), ethyl acetate, ethyl acetate:methanol (1:1) and methanol, respectively. The dried *n*-hexa­ne:ethyl­acetate (1:1) fraction was re-chromatographed by column chromatography over silica gel using increasing proportions of ethyl acetate in *n*-hexane (starting from 100% *n*-hexa­ne) as eluents to afford twelve sub-fractions. One sub-fraction, CP93-123 (6 g), obtained after elution from *n*-hexa­ne:ethyl acetate (9:1), was re-fractionated on silica gel with *n*-hexa­ne:ethyl acetate (100:0 to 80:20) to give three sub fractions (CP93-123-A, -B and -C). The sub fraction CP93-123A was suspended in *n*-hexa­ne:ethyl acetate (97:3). A white crystalline product was obtained, which was filtered and dried to give the title compound (yield 74 mg, 3.7 × 10^−4^%). Single crystals of the title compound were obtained by slow evaporation of an ethanol solution at 296 K.

## Data collection and Refinement   

Crystal data, refinement results are summarized in Table 2 ▸. All H atoms were placed geometrically (C—H = 0.95–1.00 Å and O—H = 0.84 Å) and were refined as riding with *U*_iso_(H) = 1.2*U*_eq_(C) or 1.5*U*_eq_(O). Since a partial racemic twin of the crystal was suggested from a Flack parameter of 0.17 (7) (Parsons *et al.*, 2013 ▸), a twin treatment was adopted in the final refinement. The BASF parameter refined to 0.0 (2). It is, therefore, uncertain whether the crystal used was an inversion twin or not.

## Supplementary Material

Crystal structure: contains datablock(s) global, I. DOI: 10.1107/S2056989018002499/is5486sup1.cif

Structure factors: contains datablock(s) I. DOI: 10.1107/S2056989018002499/is5486Isup2.hkl

Supporting information file. DOI: 10.1107/S2056989018002499/is5486Isup3.cml

CCDC reference: 1823530

Additional supporting information:  crystallographic information; 3D view; checkCIF report

## Figures and Tables

**Table 1:** Hydrogen-bond geometry (Å, °) *Cg*1 is the centroid of the O2/C12–C16 furan ring.

*D*—H⋯*A*	*D*—H	H⋯*A*	*D*⋯*A*	*D*—H⋯*A*
O5—H5⋯O2^i^	0.84	2.16	2.924 (2)	151
C3—H3*A*⋯*Cg*1^ii^	0.98	2.94	3.896 (3)	163
C17—H17*C*⋯O3	0.98	2.40	3.091 (3)	127

Symmetry codes: (i) 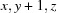; (ii) 
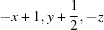
.

**Table 2:** Experimental details

Crystal data	
Chemical formula	C_29_H_36_O_5_
*M*_r_	464.58
Crystal system, space group	Monoclinic, *P*2_1_
Temperature (K)	100
*a*, *b*, *c* (Å)	12.1129 (3), 7.8973 (2), 12.9253 (3)
β (°)	94.930 (1)
*V* (Å^3^)	1231.85 (5)
*Z*	2
Radiation type	Cu *K*α
μ (mm^−1^)	0.67
Crystal size (mm)	0.17 × 0.13 × 0.06

Data collection	
Diffractometer	Bruker APEXII CCD
No. of measured, independent and observed [*I* > 2σ(*I*)] reflections	21754, 4526, 4183
*R*_int_	0.049
(sin θ/λ)_max_ (Å^−1^)	0.602

Refinement	
*R*[*F* ^2^ > 2σ(*F* ^2^)], *wR*(*F* ^2^), *S*	0.035, 0.085, 1.00
No. of reflections	4526
No. of parameters	314
No. of restraints	1
H-atom treatment	H-atom parameters constrained
Δρ_max_, Δρ_min_ (e Å^−3^)	0.19, −0.21
Absolute structure	Refined as an inversion twin
Absolute structure parameter	0.0 (2)

Computer programs: *APEX2* and *SAINT* (Bruker, 2000 ▸), *SHELXT2014* (Sheldrick, 2015*a* ▸), *SHELXL2016* (Sheldrick, 2015*b* ▸), *ORTEP-3 for Windows* (Farrugia, 2012 ▸) and *SHELXTL* (Sheldrick, 2008 ▸).

## supplementary crystallographic information

### Crystal data

**Table:**

C_29_H_36_O_5_	*F*(000) = 500
*M**_r_* = 464.58	*D*_x_ = 1.253 Mg m^−^^3^
Monoclinic, *P*2_1_	Cu *K*α radiation, λ = 1.54178 Å
*a* = 12.1129 (3) Å	Cell parameters from 9975 reflections
*b* = 7.8973 (2) Å	θ = 3.4–68.3°
*c* = 12.9253 (3) Å	µ = 0.67 mm^−^^1^
β = 94.930 (1)°	*T* = 100 K
*V* = 1231.85 (5) Å^3^	Plate, colourless
*Z* = 2	0.17 × 0.13 × 0.06 mm

### Data collection

**Table:**

Bruker APEXII CCD diffractometer	*R*_int_ = 0.049
φ and ω scans	θ_max_ = 68.3°, θ_min_ = 3.4°
21754 measured reflections	*h* = −14→14
4526 independent reflections	*k* = −9→9
4183 reflections with *I* > 2σ(*I*)	*l* = −15→15

### Refinement

**Table:**

Refinement on *F*^2^	Hydrogen site location: inferred from neighbouring sites
Least-squares matrix: full	H-atom parameters constrained
*R*[*F*^2^ > 2σ(*F*^2^)] = 0.035	*w* = 1/[σ^2^(*F*_o_^2^) + (0.0501*P*)^2^ + 0.2131*P*] where *P* = (*F*_o_^2^ + 2*F*_c_^2^)/3
*wR*(*F*^2^) = 0.085	(Δ/σ)_max_ < 0.001
*S* = 1.00	Δρ_max_ = 0.19 e Å^−^^3^
4526 reflections	Δρ_min_ = −0.21 e Å^−^^3^
314 parameters	Absolute structure: Refined as an inversion twin
1 restraint	Absolute structure parameter: 0.0 (2)

### Special details

**Table:**

Experimental. ^1^H-NMR (400 MHz C_3_D_6_O): 7.71(m), 7.62(m), 7.41(m), 7.41(m), 7.29(d, J = 1.6 Hz), 6.58(d, J = 16 Hz), 6.23(d,J = 1.6Hz), 5.65(d, J = 4Hz), 4.28(m), 3.04(q, J = 12,6.4 Hz), 2.48(m), 2.48(m), 1.97(m), 1.86(T, J = 12.8), 1.02(m), 1.72(m), 1.41(m), 1.65(m), 1.40(m), 1.46(s), 1.19(s), 1.10(m), 1.04(d, J = 7.2). ^13^C-NMR (300 MHz C_3_D_6_O): 166.9, 150.23, 145.11, 141.3, 135.5, 131.1, 129.8,129.0, 120.1, 122.9, 110.5, 78.04, 74.50, 68.93, 41.88, 39.97, 39.10, 38.43, 38.02, 35.52, 28.25, 28.18, 25.81, 22.44, 19.04, 17.77, 17.45. IR: (cm^-1^) 3592.0, 3058.8, 2934.9, 2866.8, 1713.9, 1639.7, 1577.8, 1503.1, 1456.6, 1391.8, 1310.3, 1280.8, 1168.4, 1058.3, 1008.2, 979.7, 909.7 863.8, 765.9, 723.1, 687.4.
Geometry. All esds (except the esd in the dihedral angle between two l.s. planes) are estimated using the full covariance matrix. The cell esds are taken into account individually in the estimation of esds in distances, angles and torsion angles; correlations between esds in cell parameters are only used when they are defined by crystal symmetry. An approximate (isotropic) treatment of cell esds is used for estimating esds involving l.s. planes.
Refinement. Refined as a 2-component inversion twin.

### Fractional atomic coordinates and isotropic or equivalent isotropic displacement parameters (Å^2^)

**Table:**

	*x*	*y*	*z*	*U*_iso_*/*U*_eq_	
O1	0.51780 (13)	0.1960 (2)	0.12545 (11)	0.0191 (4)	
H1	0.583625	0.166840	0.119366	0.029*	
O2	0.22809 (14)	−0.4687 (2)	−0.01284 (13)	0.0223 (4)	
O3	0.31947 (13)	0.1907 (2)	0.31852 (11)	0.0176 (3)	
O4	0.28626 (16)	0.4678 (2)	0.34903 (15)	0.0300 (4)	
O5	0.19074 (13)	0.2782 (2)	0.14554 (12)	0.0190 (4)	
H5	0.187516	0.371232	0.113926	0.029*	
C1	0.5738 (2)	−0.1463 (3)	0.19336 (19)	0.0223 (5)	
H1A	0.563465	−0.270610	0.191241	0.027*	
H1B	0.596970	−0.110358	0.125098	0.027*	
C2	0.6658 (2)	−0.1033 (4)	0.2771 (2)	0.0281 (6)	
H2A	0.646674	−0.150083	0.344371	0.034*	
H2B	0.735565	−0.157536	0.259730	0.034*	
C3	0.6840 (2)	0.0864 (4)	0.2882 (2)	0.0266 (6)	
H3A	0.711327	0.130558	0.223380	0.032*	
H3B	0.742076	0.107536	0.345374	0.032*	
C4	0.57779 (19)	0.1850 (3)	0.31052 (18)	0.0203 (5)	
C5	0.48332 (19)	0.1330 (3)	0.22378 (17)	0.0159 (5)	
C6	0.37500 (19)	0.2335 (3)	0.22738 (17)	0.0157 (5)	
H6	0.392800	0.357187	0.229305	0.019*	
C7	0.29530 (18)	0.1976 (3)	0.13233 (16)	0.0150 (5)	
H7	0.326559	0.247100	0.069884	0.018*	
C8	0.27205 (19)	0.0105 (3)	0.11248 (17)	0.0144 (5)	
H8	0.232224	−0.032713	0.171772	0.017*	
C9	0.38119 (18)	−0.0920 (3)	0.11139 (16)	0.0141 (5)	
H9	0.419571	−0.052008	0.050411	0.017*	
C10	0.46211 (19)	−0.0615 (3)	0.21063 (17)	0.0162 (5)	
C11	0.35761 (19)	−0.2846 (3)	0.09546 (18)	0.0195 (5)	
H11A	0.421988	−0.339792	0.067248	0.023*	
H11B	0.346108	−0.337965	0.163002	0.023*	
C12	0.25746 (19)	−0.3084 (3)	0.02252 (17)	0.0174 (5)	
C13	0.1808 (2)	−0.1948 (3)	−0.01287 (18)	0.0169 (5)	
C14	0.19243 (19)	−0.0105 (3)	0.01221 (18)	0.0170 (5)	
H14	0.117939	0.033202	0.027082	0.020*	
C15	0.09710 (19)	−0.2868 (3)	−0.07485 (19)	0.0214 (5)	
H15	0.031927	−0.241267	−0.110517	0.026*	
C16	0.1288 (2)	−0.4498 (3)	−0.0727 (2)	0.0240 (5)	
H16	0.088574	−0.539445	−0.107408	0.029*	
C17	0.5565 (2)	0.1516 (3)	0.42506 (18)	0.0252 (6)	
H17A	0.612996	0.209595	0.470912	0.038*	
H17B	0.559908	0.029565	0.438823	0.038*	
H17C	0.482962	0.194371	0.438008	0.038*	
C18	0.6012 (2)	0.3760 (3)	0.3034 (2)	0.0274 (6)	
H18A	0.540690	0.439604	0.330854	0.041*	
H18B	0.606793	0.407338	0.230669	0.041*	
H18C	0.671040	0.402637	0.344118	0.041*	
C19	0.4136 (2)	−0.1444 (3)	0.30596 (17)	0.0194 (5)	
H19A	0.444707	−0.088795	0.369703	0.029*	
H19B	0.432678	−0.265020	0.308648	0.029*	
H19C	0.332817	−0.131699	0.299626	0.029*	
C20	0.2289 (2)	0.0857 (3)	−0.08241 (18)	0.0218 (5)	
H20A	0.225048	0.207822	−0.069787	0.033*	
H20B	0.179661	0.056395	−0.143969	0.033*	
H20C	0.305132	0.054200	−0.093813	0.033*	
C21	0.2732 (2)	0.3200 (3)	0.36808 (18)	0.0204 (5)	
C22	0.2066 (2)	0.2519 (4)	0.44928 (18)	0.0221 (6)	
H22	0.205628	0.133757	0.462953	0.026*	
C23	0.1479 (2)	0.3580 (4)	0.50308 (18)	0.0235 (6)	
H23	0.153196	0.474847	0.486769	0.028*	
C24	0.0756 (2)	0.3140 (4)	0.58504 (19)	0.0242 (6)	
C25	0.0608 (2)	0.1487 (4)	0.61904 (19)	0.0283 (6)	
H25	0.098725	0.058097	0.589145	0.034*	
C26	−0.0092 (2)	0.1158 (4)	0.6966 (2)	0.0329 (7)	
H26	−0.019633	0.002787	0.719015	0.039*	
C27	−0.0638 (2)	0.2486 (5)	0.7412 (2)	0.0361 (8)	
H27	−0.111276	0.226126	0.794396	0.043*	
C28	−0.0492 (2)	0.4132 (4)	0.7086 (2)	0.0335 (7)	
H28	−0.086528	0.503687	0.739264	0.040*	
C29	0.0199 (2)	0.4457 (4)	0.6310 (2)	0.0290 (6)	
H29	0.029684	0.558944	0.608671	0.035*	

### Atomic displacement parameters (Å^2^)

**Table:**

	*U*^11^	*U*^22^	*U*^33^	*U*^12^	*U*^13^	*U*^23^
O1	0.0156 (8)	0.0276 (9)	0.0152 (8)	0.0023 (7)	0.0070 (6)	0.0042 (7)
O2	0.0289 (9)	0.0153 (9)	0.0219 (9)	0.0013 (7)	−0.0027 (7)	0.0011 (7)
O3	0.0202 (8)	0.0201 (8)	0.0138 (7)	0.0030 (7)	0.0085 (6)	0.0012 (7)
O4	0.0332 (11)	0.0233 (10)	0.0361 (10)	−0.0011 (8)	0.0176 (9)	−0.0085 (8)
O5	0.0184 (9)	0.0168 (8)	0.0224 (8)	0.0066 (7)	0.0055 (7)	0.0035 (7)
C1	0.0210 (13)	0.0254 (13)	0.0201 (12)	0.0091 (10)	−0.0012 (10)	−0.0019 (10)
C2	0.0227 (13)	0.0350 (15)	0.0256 (13)	0.0130 (12)	−0.0047 (11)	−0.0087 (12)
C3	0.0183 (12)	0.0369 (16)	0.0238 (13)	0.0043 (11)	−0.0027 (10)	−0.0051 (11)
C4	0.0183 (11)	0.0245 (13)	0.0179 (11)	0.0006 (11)	0.0010 (9)	−0.0016 (11)
C5	0.0167 (11)	0.0210 (12)	0.0107 (10)	0.0020 (9)	0.0054 (9)	0.0020 (9)
C6	0.0185 (11)	0.0159 (12)	0.0137 (10)	−0.0002 (9)	0.0078 (9)	0.0026 (9)
C7	0.0151 (11)	0.0157 (11)	0.0149 (10)	0.0051 (10)	0.0060 (9)	0.0032 (10)
C8	0.0152 (11)	0.0164 (12)	0.0122 (10)	0.0021 (9)	0.0046 (9)	0.0040 (9)
C9	0.0161 (11)	0.0156 (11)	0.0111 (10)	0.0038 (9)	0.0039 (8)	0.0015 (9)
C10	0.0189 (12)	0.0168 (12)	0.0131 (10)	0.0042 (9)	0.0022 (9)	0.0004 (9)
C11	0.0240 (12)	0.0191 (12)	0.0151 (10)	0.0053 (11)	−0.0004 (9)	0.0017 (10)
C12	0.0228 (12)	0.0160 (11)	0.0141 (10)	0.0004 (10)	0.0049 (9)	0.0014 (10)
C13	0.0164 (11)	0.0187 (12)	0.0162 (11)	−0.0003 (9)	0.0054 (9)	0.0029 (9)
C14	0.0138 (11)	0.0172 (12)	0.0201 (11)	0.0034 (9)	0.0027 (9)	0.0017 (9)
C15	0.0161 (11)	0.0239 (13)	0.0241 (12)	−0.0018 (10)	0.0007 (9)	0.0031 (11)
C16	0.0242 (13)	0.0218 (13)	0.0254 (13)	−0.0047 (11)	−0.0021 (10)	0.0014 (11)
C17	0.0281 (14)	0.0306 (15)	0.0165 (11)	0.0030 (11)	−0.0007 (10)	−0.0044 (10)
C18	0.0245 (13)	0.0267 (15)	0.0307 (14)	−0.0021 (11)	0.0010 (11)	−0.0034 (11)
C19	0.0291 (13)	0.0169 (12)	0.0121 (10)	0.0043 (10)	0.0014 (10)	0.0024 (9)
C20	0.0290 (13)	0.0179 (12)	0.0175 (12)	−0.0022 (11)	−0.0043 (10)	0.0042 (9)
C21	0.0181 (12)	0.0257 (14)	0.0178 (12)	0.0002 (10)	0.0038 (10)	−0.0049 (10)
C22	0.0197 (12)	0.0314 (15)	0.0154 (11)	0.0009 (10)	0.0030 (10)	−0.0026 (10)
C23	0.0179 (12)	0.0354 (15)	0.0172 (12)	−0.0010 (11)	0.0014 (10)	−0.0058 (11)
C24	0.0134 (12)	0.0434 (17)	0.0157 (11)	−0.0003 (11)	0.0001 (10)	−0.0073 (11)
C25	0.0214 (13)	0.0448 (18)	0.0186 (12)	0.0038 (11)	0.0012 (10)	−0.0045 (12)
C26	0.0265 (14)	0.0487 (18)	0.0235 (13)	−0.0032 (13)	0.0030 (11)	0.0043 (13)
C27	0.0252 (14)	0.064 (2)	0.0198 (13)	0.0011 (14)	0.0094 (11)	−0.0011 (13)
C28	0.0213 (13)	0.054 (2)	0.0265 (14)	0.0029 (13)	0.0071 (11)	−0.0092 (14)
C29	0.0194 (13)	0.0432 (18)	0.0247 (13)	0.0005 (12)	0.0045 (11)	−0.0060 (13)

### Geometric parameters (Å, º)

**Table:**

O1—C5	1.459 (3)	C11—H11B	0.9900
O1—H1	0.8400	C12—C13	1.343 (3)
O2—C16	1.381 (3)	C13—C15	1.435 (3)
O2—C12	1.383 (3)	C13—C14	1.495 (3)
O3—C21	1.352 (3)	C14—C20	1.536 (3)
O3—C6	1.446 (3)	C14—H14	1.0000
O4—C21	1.206 (3)	C15—C16	1.343 (4)
O5—C7	1.441 (3)	C15—H15	0.9500
O5—H5	0.8400	C16—H16	0.9500
C1—C2	1.524 (3)	C17—H17A	0.9800
C1—C10	1.543 (3)	C17—H17B	0.9800
C1—H1A	0.9900	C17—H17C	0.9800
C1—H1B	0.9900	C18—H18A	0.9800
C2—C3	1.520 (4)	C18—H18B	0.9800
C2—H2A	0.9900	C18—H18C	0.9800
C2—H2B	0.9900	C19—H19A	0.9800
C3—C4	1.552 (3)	C19—H19B	0.9800
C3—H3A	0.9900	C19—H19C	0.9800
C3—H3B	0.9900	C20—H20A	0.9800
C4—C18	1.539 (4)	C20—H20B	0.9800
C4—C17	1.547 (3)	C20—H20C	0.9800
C4—C5	1.586 (3)	C21—C22	1.479 (3)
C5—C6	1.538 (3)	C22—C23	1.333 (3)
C5—C10	1.564 (3)	C22—H22	0.9500
C6—C7	1.522 (3)	C23—C24	1.472 (3)
C6—H6	1.0000	C23—H23	0.9500
C7—C8	1.522 (3)	C24—C25	1.394 (4)
C7—H7	1.0000	C24—C29	1.400 (4)
C8—C9	1.551 (3)	C25—C26	1.392 (4)
C8—C14	1.556 (3)	C25—H25	0.9500
C8—H8	1.0000	C26—C27	1.392 (4)
C9—C11	1.558 (3)	C26—H26	0.9500
C9—C10	1.565 (3)	C27—C28	1.383 (5)
C9—H9	1.0000	C27—H27	0.9500
C10—C19	1.555 (3)	C28—C29	1.385 (4)
C11—C12	1.483 (3)	C28—H28	0.9500
C11—H11A	0.9900	C29—H29	0.9500
			
C5—O1—H1	109.5	C13—C12—O2	110.4 (2)
C16—O2—C12	105.83 (18)	C13—C12—C11	129.5 (2)
C21—O3—C6	116.78 (18)	O2—C12—C11	120.0 (2)
C7—O5—H5	109.5	C12—C13—C15	106.7 (2)
C2—C1—C10	113.4 (2)	C12—C13—C14	121.8 (2)
C2—C1—H1A	108.9	C15—C13—C14	131.5 (2)
C10—C1—H1A	108.9	C13—C14—C20	109.7 (2)
C2—C1—H1B	108.9	C13—C14—C8	108.94 (19)
C10—C1—H1B	108.9	C20—C14—C8	114.22 (19)
H1A—C1—H1B	107.7	C13—C14—H14	107.9
C3—C2—C1	112.2 (2)	C20—C14—H14	107.9
C3—C2—H2A	109.2	C8—C14—H14	107.9
C1—C2—H2A	109.2	C16—C15—C13	106.7 (2)
C3—C2—H2B	109.2	C16—C15—H15	126.7
C1—C2—H2B	109.2	C13—C15—H15	126.7
H2A—C2—H2B	107.9	C15—C16—O2	110.4 (2)
C2—C3—C4	113.4 (2)	C15—C16—H16	124.8
C2—C3—H3A	108.9	O2—C16—H16	124.8
C4—C3—H3A	108.9	C4—C17—H17A	109.5
C2—C3—H3B	108.9	C4—C17—H17B	109.5
C4—C3—H3B	108.9	H17A—C17—H17B	109.5
H3A—C3—H3B	107.7	C4—C17—H17C	109.5
C18—C4—C17	105.7 (2)	H17A—C17—H17C	109.5
C18—C4—C3	108.7 (2)	H17B—C17—H17C	109.5
C17—C4—C3	107.6 (2)	C4—C18—H18A	109.5
C18—C4—C5	109.7 (2)	C4—C18—H18B	109.5
C17—C4—C5	117.6 (2)	H18A—C18—H18B	109.5
C3—C4—C5	107.32 (19)	C4—C18—H18C	109.5
O1—C5—C6	99.16 (17)	H18A—C18—H18C	109.5
O1—C5—C10	107.26 (18)	H18B—C18—H18C	109.5
C6—C5—C10	112.19 (19)	C10—C19—H19A	109.5
O1—C5—C4	106.52 (18)	C10—C19—H19B	109.5
C6—C5—C4	114.24 (19)	H19A—C19—H19B	109.5
C10—C5—C4	115.66 (18)	C10—C19—H19C	109.5
O3—C6—C7	107.87 (17)	H19A—C19—H19C	109.5
O3—C6—C5	111.22 (17)	H19B—C19—H19C	109.5
C7—C6—C5	111.28 (18)	C14—C20—H20A	109.5
O3—C6—H6	108.8	C14—C20—H20B	109.5
C7—C6—H6	108.8	H20A—C20—H20B	109.5
C5—C6—H6	108.8	C14—C20—H20C	109.5
O5—C7—C8	107.28 (18)	H20A—C20—H20C	109.5
O5—C7—C6	108.97 (18)	H20B—C20—H20C	109.5
C8—C7—C6	114.34 (19)	O4—C21—O3	124.5 (2)
O5—C7—H7	108.7	O4—C21—C22	125.8 (2)
C8—C7—H7	108.7	O3—C21—C22	109.6 (2)
C6—C7—H7	108.7	C23—C22—C21	119.4 (2)
C7—C8—C9	111.26 (18)	C23—C22—H22	120.3
C7—C8—C14	109.65 (18)	C21—C22—H22	120.3
C9—C8—C14	113.85 (17)	C22—C23—C24	127.1 (3)
C7—C8—H8	107.3	C22—C23—H23	116.4
C9—C8—H8	107.3	C24—C23—H23	116.4
C14—C8—H8	107.3	C25—C24—C29	118.9 (2)
C8—C9—C11	111.34 (18)	C25—C24—C23	123.3 (2)
C8—C9—C10	112.74 (17)	C29—C24—C23	117.9 (3)
C11—C9—C10	110.70 (18)	C26—C25—C24	120.3 (3)
C8—C9—H9	107.3	C26—C25—H25	119.8
C11—C9—H9	107.3	C24—C25—H25	119.8
C10—C9—H9	107.3	C27—C26—C25	119.9 (3)
C1—C10—C19	109.14 (19)	C27—C26—H26	120.1
C1—C10—C5	107.69 (19)	C25—C26—H26	120.1
C19—C10—C5	113.40 (18)	C28—C27—C26	120.3 (3)
C1—C10—C9	108.06 (17)	C28—C27—H27	119.9
C19—C10—C9	109.40 (18)	C26—C27—H27	119.9
C5—C10—C9	109.01 (18)	C27—C28—C29	119.8 (3)
C12—C11—C9	109.77 (19)	C27—C28—H28	120.1
C12—C11—H11A	109.7	C29—C28—H28	120.1
C9—C11—H11A	109.7	C28—C29—C24	120.9 (3)
C12—C11—H11B	109.7	C28—C29—H29	119.6
C9—C11—H11B	109.7	C24—C29—H29	119.6
H11A—C11—H11B	108.2		
			
C10—C1—C2—C3	−56.4 (3)	C4—C5—C10—C9	−170.95 (17)
C1—C2—C3—C4	56.2 (3)	C8—C9—C10—C1	−171.20 (19)
C2—C3—C4—C18	−171.4 (2)	C11—C9—C10—C1	63.3 (2)
C2—C3—C4—C17	74.6 (3)	C8—C9—C10—C19	70.1 (2)
C2—C3—C4—C5	−52.8 (3)	C11—C9—C10—C19	−55.4 (2)
C18—C4—C5—O1	52.4 (2)	C8—C9—C10—C5	−54.4 (2)
C17—C4—C5—O1	173.2 (2)	C11—C9—C10—C5	−179.89 (18)
C3—C4—C5—O1	−65.5 (2)	C8—C9—C11—C12	36.7 (2)
C18—C4—C5—C6	−56.0 (2)	C10—C9—C11—C12	162.98 (17)
C17—C4—C5—C6	64.8 (3)	C16—O2—C12—C13	0.1 (2)
C3—C4—C5—C6	−173.9 (2)	C16—O2—C12—C11	176.0 (2)
C18—C4—C5—C10	171.51 (19)	C9—C11—C12—C13	−12.6 (3)
C17—C4—C5—C10	−67.7 (3)	C9—C11—C12—O2	172.36 (19)
C3—C4—C5—C10	53.6 (3)	O2—C12—C13—C15	−0.1 (3)
C21—O3—C6—C7	−98.4 (2)	C11—C12—C13—C15	−175.5 (2)
C21—O3—C6—C5	139.3 (2)	O2—C12—C13—C14	−179.1 (2)
O1—C5—C6—O3	178.25 (17)	C11—C12—C13—C14	5.5 (4)
C10—C5—C6—O3	65.3 (2)	C12—C13—C14—C20	103.5 (2)
C4—C5—C6—O3	−68.9 (2)	C15—C13—C14—C20	−75.2 (3)
O1—C5—C6—C7	58.0 (2)	C12—C13—C14—C8	−22.2 (3)
C10—C5—C6—C7	−55.0 (2)	C15—C13—C14—C8	159.1 (2)
C4—C5—C6—C7	170.81 (19)	C7—C8—C14—C13	173.58 (18)
O3—C6—C7—O5	50.8 (2)	C9—C8—C14—C13	48.2 (2)
C5—C6—C7—O5	173.09 (18)	C7—C8—C14—C20	50.6 (2)
O3—C6—C7—C8	−69.2 (2)	C9—C8—C14—C20	−74.8 (3)
C5—C6—C7—C8	53.1 (2)	C12—C13—C15—C16	0.0 (3)
O5—C7—C8—C9	−172.61 (16)	C14—C13—C15—C16	178.9 (2)
C6—C7—C8—C9	−51.7 (2)	C13—C15—C16—O2	0.0 (3)
O5—C7—C8—C14	60.5 (2)	C12—O2—C16—C15	−0.1 (3)
C6—C7—C8—C14	−178.51 (17)	C6—O3—C21—O4	−9.1 (3)
C7—C8—C9—C11	177.74 (18)	C6—O3—C21—C22	171.78 (18)
C14—C8—C9—C11	−57.7 (2)	O4—C21—C22—C23	5.2 (4)
C7—C8—C9—C10	52.6 (2)	O3—C21—C22—C23	−175.7 (2)
C14—C8—C9—C10	177.15 (19)	C21—C22—C23—C24	178.9 (2)
C2—C1—C10—C19	−70.0 (3)	C22—C23—C24—C25	1.2 (4)
C2—C1—C10—C5	53.5 (3)	C22—C23—C24—C29	−179.2 (2)
C2—C1—C10—C9	171.1 (2)	C29—C24—C25—C26	0.7 (4)
O1—C5—C10—C1	64.7 (2)	C23—C24—C25—C26	−179.7 (2)
C6—C5—C10—C1	172.59 (17)	C24—C25—C26—C27	−0.7 (4)
C4—C5—C10—C1	−53.9 (2)	C25—C26—C27—C28	0.3 (4)
O1—C5—C10—C19	−174.41 (18)	C26—C27—C28—C29	0.1 (4)
C6—C5—C10—C19	−66.6 (2)	C27—C28—C29—C24	−0.1 (4)
C4—C5—C10—C19	66.9 (3)	C25—C24—C29—C28	−0.3 (4)
O1—C5—C10—C9	−52.3 (2)	C23—C24—C29—C28	−179.9 (2)
C6—C5—C10—C9	55.6 (2)		

### Hydrogen-bond geometry (Å, º)

*Cg*1 is the centroid of the O2/C12–C16 furan ring.

**Table:**

*D*—H···*A*	*D*—H	H···*A*	*D*···*A*	*D*—H···*A*
O5—H5···O2^i^	0.84	2.16	2.924 (2)	151
C3—H3*A*···*Cg*1^ii^	0.98	2.94	3.896 (3)	163
C17—H17*C*···O3	0.98	2.40	3.091 (3)	127

Symmetry codes: (i) *x*, *y*+1, *z*; (ii) −*x*+1, *y*+1/2, −*z*.
